# Transcriptional Changes in Canine Distemper Virus-Induced Demyelinating Leukoencephalitis Favor a Biphasic Mode of Demyelination

**DOI:** 10.1371/journal.pone.0095917

**Published:** 2014-04-22

**Authors:** Reiner Ulrich, Christina Puff, Konstantin Wewetzer, Arno Kalkuhl, Ulrich Deschl, Wolfgang Baumgärtner

**Affiliations:** 1 Department of Pathology, University of Veterinary Medicine Hannover, Hannover, Germany; 2 Department of Functional and Applied Anatomy, Hannover Medical School, Hannover, Germany; 3 Department of Non-Clinical Drug Safety, Boehringer Ingelheim Pharma GmbH&Co KG, Biberach (Riβ), Germany; 4 Center of Systems Neuroscience, Hannover, Germany; Virginia Polytechnic Institute and State University, United States of America

## Abstract

*Canine distemper virus (CDV)*-induced demyelinating leukoencephalitis in dogs (*Canis familiaris*) is suggested to represent a naturally occurring translational model for subacute sclerosing panencephalitis and multiple sclerosis in humans. The aim of this study was a hypothesis-free microarray analysis of the transcriptional changes within cerebellar specimens of five cases of acute, six cases of subacute demyelinating, and three cases of chronic demyelinating and inflammatory CDV leukoencephalitis as compared to twelve non-infected control dogs. Frozen cerebellar specimens were used for analysis of histopathological changes including demyelination, transcriptional changes employing microarrays, and presence of CDV nucleoprotein RNA and protein using microarrays, RT-qPCR and immunohistochemistry. Microarray analysis revealed 780 differentially expressed probe sets. The dominating change was an up-regulation of genes related to the innate and the humoral immune response, and less distinct the cytotoxic T-cell-mediated immune response in all subtypes of CDV leukoencephalitis as compared to controls. Multiple myelin genes including *myelin basic protein* and *proteolipid protein* displayed a selective down-regulation in subacute CDV leukoencephalitis, suggestive of an oligodendrocyte dystrophy. In contrast, a marked up-regulation of multiple *immunoglobulin-like expressed sequence tags* and the *delta polypeptide of the CD3 antigen* was observed in chronic CDV leukoencephalitis, in agreement with the hypothesis of an immune-mediated demyelination in the late inflammatory phase of the disease. Analysis of pathways intimately linked to demyelination as determined by morphometry employing correlation-based Gene Set Enrichment Analysis highlighted the pathomechanistic importance of up-regulated genes comprised by the gene ontology terms “viral replication” and “humoral immune response” as well as down-regulated genes functionally related to “metabolite and energy generation”.

## Introduction

*Canine distemper virus* (CDV) is a morbillivirus of the family *Paramyxoviridae* and the etiologic agent of distemper in dogs (*Canis familiaris*). Distemper is a naturally and worldwide-occuring, highly contagious systemic virus infection of dogs as well as various other carnivores [1]. The mortality rate can be up to 50% but is often much lower [2]. The route of infection is mainly oronasally followed by a first viremic spread of CDV to lymphoid tissues resulting in lymphopenia and immunosuppression [3]. Subsequently, depending on the virulence of the respective CDV strain, the host’s age and immune status, dogs may either initiate a robust humoral immune response and recover or may develop a second viremic spread to multiple organs and tissues [4], [5], [6]. These dogs show various pathologic alterations including thymic atrophy, conjunctivitis, rhinitis, interstitial pneumonia, encephalitis, gastroenteritis, pustular dermatitis and hyperkeratosis of the foot pads and nose [3], [7], [8], [9]. Dogs may either succumb to CDV infection during this phase or recover. However, some dogs may develop a chronically persistent or late-onset encephalitis [10], [11].

CDV-induced encephalitis frequently manifests as an acute, subacute or chronic leukoencephalitis, and rarely as a polioencephalitis [12], [13]. CDV leukoencephalitis lesions are found mainly periventricularly in the cerebellum, optic tract, medulla oblongata, cerebral peduncle, spinal cord, and less frequently in the subcortical white matter, fornix, capsula interna and corpus callosum [12], [14]. Based on histopathological findings in experimentally-induced CDV leukoencephalitis in dogs, central nervous system (CNS) lesions can be classified into distinct subtypes presumably developing in sequential order [14], [15]. Briefly, the hallmark of acute lesions are focal accumulations of CDV antigen-positive glial cells, eventually accompanied by vacuolation of the white matter, and mild gliosis [14], [16], [17]. This type of lesions has been observed 16–24 days after experimental CDV-infection [15], [16], [18], [19]. Subacute lesions with demyelination but without inflammation may occur 24–32 days post infection [15], [16], [18], [19], [20]. Subacute to chronic lesions with demyelination and inflammation are characterized by reduced numbers of CDV antigen-positive cells and distinct perivascular lymphohistioplasmacytic cuffs of several layers of thickness [14], [16], [17]. Following experimental infections, this type of lesions occurred after a minimum of 29–63 days post infection [5], [15], [16], [18], [19], [20], Overall the correlation between morphological changes and assumed time point of infection remains a vague supposition in cases of spontaneously occurring CDV leukoencephalitis, since multiple factors including virus strain, immune status and age of the host as well as various environmental parameters influence virus spread and disease progression [4], [5], [6].

CDV leukoencephalitis in dogs has been suggested as a spontaneously-occuring viral model to study the pathogenesis of demyelination in subacute sclerosing panencephalitis and multiple sclerosis in humans [21], [22]. Suggested mechanisms mediating primary demyelination in CDV leukoencephalitis include virus-induced damage of oligodendrocytes [19], [23], bystander damage due to activated microglia/macrophages releasing myelinotoxic reactive oxygen species and proteolytic enzymes such as matrix metalloproteinases [24], [25], [26], activated astrocytes and microglia/macrophages releasing tumor necrosis factor-α (TNFα) [27], and humoral and cell-mediated autoimmunity [28], [29], [30], [31].

In order to shed more light on the molecular pathogenesis of CDV leukoencephalitis, the aims of the present investigation were 1.) to perform a classic assumption-free microarray analysis of transcriptional changes in CDV leukoencephalitis, and 2.) to identify pathways intimately associated with demyelination by combining findings obtained by histological analysis of brain sections of different subtypes of CDV leukoencephalitis with respective microarray data employing correlation-based Gene Set Enrichment Analysis.

## Materials and Methods

### Ethics Statement

This study was conducted in accordance with the German Animal Welfare Act. The authors confirm that no animals were infected or sacrificed for the purpose of this retrospective pathological case-control study. This study is not an animal experiment since all animals were dead at the time of submission for necropsy in order to investigate the causes of death and disease. All tissues used in this study were collected by one of the authors (WB) during his work at the diagnostic pathology services of the Department of Pathology, University of Veterinary Medicine Hannover, and the Institute of Veterinary Pathology, Justus-Liebig-University Giessen, and most animals were used in previous publications [32], [33]. All dog owners provided written consent for the dogs’ tissues to be collected and used for research purposes.

### Experimental Design

Age, breed and gender of the dogs are presented in Table 1. Twelve control animals (group 1) covering a comparable range of variation with respect to gender, age and breed as CDV-infected dogs, and lacking morphological evidence of CNS disease were selected from the departmental archives. The 14 CDV-infected dogs suffered from spontaneously-occurring and immunohistologically-confirmed CDV leukoencephalitis. For the current study, only specimens of cerebellum and adjacent brain stem unequivocally displaying focal or multifocal lesions of a single subtype of CDV leukoencephalitis within each individual dog were selected. Based on histopathological findings, dogs were classified into acute CDV leukoencephalitis (group 2, N = 5), subacute CDV leukoencephalitis with demyelination but without inflammation (group 3, N = 6), and (subacute to) chronic CDV leukoencephalitis with demyelination and inflammation (group 4, N = 3) according to previously described criteria [17], [33].

**Table 1:** Experimental design and signalement of dogs with CDV leukoencephalitis and controls.

Group ID	Animal ID	Breed	Gender	Age (month)	Body weight (kg)
1	V723-06	Beagle	m	6	8.25
1	V725-06	Beagle	f	6	10.80
1	V776-06	Beagle	f	5	6.50
1	V777-06	Beagle	m	5	7.45
1	V239-05	Beagle	m	19	n.d.
1	V255-05	Beagle	m	20	n.d.
1	V256-05	Beagle	f	17	n.d.
1	V259-05	Beagle	f	17	n.d.
1	S1486-98	Cavalier King Charles Spaniel	f	8	3.00
1	S1497-98	Rhodesian Ridgeback	m	8	32.00
1	S521-99	Giant Schnauzer	f	5	16.00
1	S1863-98	Yorkshire Terrier	f	3	0.75
2	S104-95	Mixed breed	m	5	5.50
2	S2548-90	Bobtail	m	6	n.d.
2	S842-04	Mixed breed	f	4	5.00
2	S997-03	Jack-Russell-Terrier	m	5	3.00
2	S580-05	Mixed breed	m	3	2.10
3	S1358-90	Terrier	f	4	4.00
3	S98-91	Mixed breed	f	6	n.d.
3	S2015-91	German sheperd	m	4	n.d.
3	S103-95	Dachshund	f	12	5.00
3	S58-05	Husky	f	7	12.80
3	S954-05	Mixed breed	m	6	13.00
4	S2936-04	Jack-Russell-Terrier	f	3	3.50
4	S1510-90	Mixed breed	m	47	n.d.
4	S2290-90	German sheperd	m	10	n.d.

Group ID: 1 = control dogs, 2 = acute CDV leukoencephalitis, 3 = subacute CDV leukoencephalitis, 4 = chronic CDV leukoencephalitis; f = female; ID = identifier; m = male; n.d. = not determined.

### Histology and Immunohistology

Cerebellar specimens were snap-frozen in Tissue-Tek O.C.T. compound (Sakura Finetek Europe B.V. Zoeterwoude, Netherlands) and ∼4 µm thick sections, contiguous to the tissue used for RNA isolation, were cut on a cryotome, mounted on SuperFrost Plus microscope slides (Menzel-Gläser, Braunschweig, Germany), and stained with hematoxylin and eosin (HE), and Luxol fast blue-cresyl violet. Immunohistological examination of serial sections was performed with the avidin-biotin-peroxidase complex (ABC) method (Vector PK-6100) and 3,3′-diaminobenzidine-tetrahydrochloride as chromogen. A polyclonal rabbit antibody specific for the CDV nucleoprotein (diluted 1∶2000, #25, kindly provided by C. Örvell, The National Bacteriological Laboratory, Stockholm, Sweden) was used to confirm CDV infection [33].

### RNA Isolation, Microarray Hybridization and Low-level Analysis

Total RNA was isolated from the frozen cerebellar specimens using the RNeasy Lipid Tissue Mini Kit (Qiagen, Hilden, Germany). The quality and integrity of the RNA was controlled using the Agilent 6000 RNA Nano kit and an Agilent Bioanalyzer 2100 (Agilent, Böblingen, Germany). 200 ng of each RNA was amplified and biotin-labeled employing the 3′IVT express kit (Affymetrix, Santa Clara, USA), and hybridized to GeneChip canine genome 2.0 arrays (Affymetrix, Santa Clara, USA) in a rotating hybridization oven at 45°C for 16 hrs. Afterwards the arrays were washed and stained with a solution containing R-phycoerythrin-streptavidin employing the Affymetrix GeneChip Fluidics Station 450 (Affymetrix, Santa Clara, USA). Scanning was performed with an Affymetrix GeneChip Scanner 3000 (Affymetrix, Santa Clara, USA). Background adjustment, quantile normalization and probe set summarization was performed using the GC-RMA algorithm (Bioconductor *gcrma* for R package, Version 2.3) [34]. Principal components analysis was performed as a quality check for outlying samples employing Babelomics [35]. MIAME compliant data sets are deposited in the ArrayExpress database (accession number: E-MEXP-3917; http://www.ebi.ac.uk/arrayexpress). The mean expression values of the spiked-in hybridization control RNAs (1.5 pM *Escherichia coli biotin synthase [bioB]*, 5 pM *Escherichia coli biotin biosynthesis protein [bioC]*, 25 pM *Escherichia coli dethiobiotin synthetase [bioD],* 100 pM *bacteriophage P1 cyclization recombinase [cre]*, Affymetrix, Santa Clara, USA) were used as comparative markers for no to low, moderate, high, or highest expression, respectively.

### Differentially Expressed Probe Sets

Differentially expressed probe sets (DEPs) were detected employing a one-factorial test using the Linear Models for Microarray Data (LIMMA) algorithm with a maximal false discovery rate (FDR) of 5% (q≤0.05) according to Benjamini and Hochberg [35], [36], followed by *post-hoc* pairwise comparisons of group 1–4 combining a statistical significance filter (LIMMA, q≤0.05) and a fold change filter (fold change ≥2.0 or ≤ −2.0). The fold change was calculated as the ratio of the inverse-transformed arithmetic means of the log_2_-transformed expression values. Down-regulations are shown as negative reciprocal values. For the hierarchical clusters we used TM4 Multi Experiment Viewer (MeV) with the individual fold change of each animal relative to the mean of all control dogs, Euclidean distance, and complete linkage [37]. Gene lists were compared and analyzed for intersections employing Venn diagrams (Oliveros, J.C. VENNY. An interactive tool for comparing lists with Venn Diagrams. http://bioinfogp.cnb.csic.es/tools/venny/index.html.).

### Annotation and Gene Ontology Information

Probe sets were annotated with canine gene symbols and gene names according to the Affymetrix annotation file (release 33; 29. October 2012). Due to non-perfect accordance of the multiple co-existing genomic identifier (ID) and nomenclature systems (official gene symbol, Unigene ID, Entrez Gene ID, etc.) genes were defined according to the integrative DAVID knowledgebase (DAVID ID) throughout this text [38]. Orthologous mouse gene symbols were retrieved employing MADGene [39]. Lists of DEPs were consolidated to lists of differentially expressed genes (DEGs) by selecting the probe set with the highest significant absolute fold change in the respective *post hoc* test.

The lists of DEPs or DEGs were checked for significantly overrepresented functional terms of the biological process category of the gene ontology database employing a modified Fisher exact test (EASE score) in DAVID 6.7 [40], [41]. Due to the generally low frequency of deposited functional gene ontology associations for the canine genes, an alternative approach employing orthologous mouse genes was used [42]. The resulting lists of gene ontology terms were agglomerated into a manageable number of ≤10 enriched biological modules employing the DAVID functional annotation clustering algorithm [43]. The enrichment score used to rank these modules according to their biological relevance is calculated as the negative log_10_ of the geometric mean of the EASE scores of all incorporated gene ontology terms.

### Gene Set Enrichment Analysis

For the identification of biological processes intimately associated with myelin loss, Gene Set Enrichment Analysis (GSEA, Version 2.0.10) was performed employing Pearson’s correlation coefficient as metric to rank the genes according to their correlation to demyelination as observed in Luxol fast blue-cresyl violet stained sections and checked for enriched gene ontology biological process terms from the Molecular Signatures Database (MSigDB) Version 3.1 [44]. The percentage of Luxol fast blue-negative white matter in relation to the total white matter on each slide was calculated as input. The microarray data set was pre-filtered for informative probe sets with a q≤0.05 in the multigroup LIMMA [45]. Orthologous and unique human gene symbols (HUGO), required as feature identifiers by Gene Set Enrichment Analysis were retrieved employing MADGene [39]. Gene sets in a size range from 4 to 10 genes and positively or negatively correlated to demyelination with a p≤0.05 were accepted as significant and ranked according to the normalized enrichment score [44].

### RT-qPCR

Reverse transcription quantitative polymerase chain reaction (RT-qPCR) was performed from the same batch of RNA that was used for the microarray analysis. Total RNA was reverse transcribed using the Omniscript kit (Qiagen, Hilden, Germany) and random primers (Promega, Mannheim, Germany). Quantitative PCR and data analysis were performed with the Brilliant SYBR-green QPCR Core Reagent Kit (Agilent, Santa Clara, CA), the Mx3005P QPCR System (Agilent, Santa Clara, CA), and calibration by an external standard curve. Used primer pairs were specific for *CDV nucleoprotein* (sense: 5′ GCTCTTGGGTTGCATGAGTT, antisense: 5′GCTGTTTCACCCATCTGTTG, amplicon size: 83 base pairs), and *glyceraldehyde-3-phosphate dehydrogenase (GAPDH)* as house-keeping gene (sense: 5′ GTCATCAACGGGAAGTCCATCTC, antisense: 5′ AACATACTCAGCACCAGCATCAC, amplicon size: 84 base pairs) [46]. The specificity of positive reactions was confirmed by melting point analysis. *CDV nucleoprotein* RNA copy numbers were normalized with a normalization factor based on *GAPDH* expression.

### Statistical Analysis

Analysis of data not otherwise specified was performed using IBM SPSS Statistics, Version 20 (IBM Corporation, Armonk, NY, USA). Used statistical procedures included the non-parametric Kruskal-Wallis test with *post-hoc* independent pairwise Mann-Whitney U-tests for the analysis of the Luxol fast blue-negative white matter area, CDV nucleoprotein immunoreactive cells, microarray and RT-qPCR data of CDV nucleoprotein RNA, and microarray data of manually selected glial cell marker genes. Generally, a p-value ≤0.05 was accepted as significant. Graphs were generated employing GrapPad Prism, Version 6.01 (GraphPad Software, La Jolla, CA, USA).

## Results

### Histological and Immunohistological changes in CDV Leukoencephalitis

**Figure 1:**
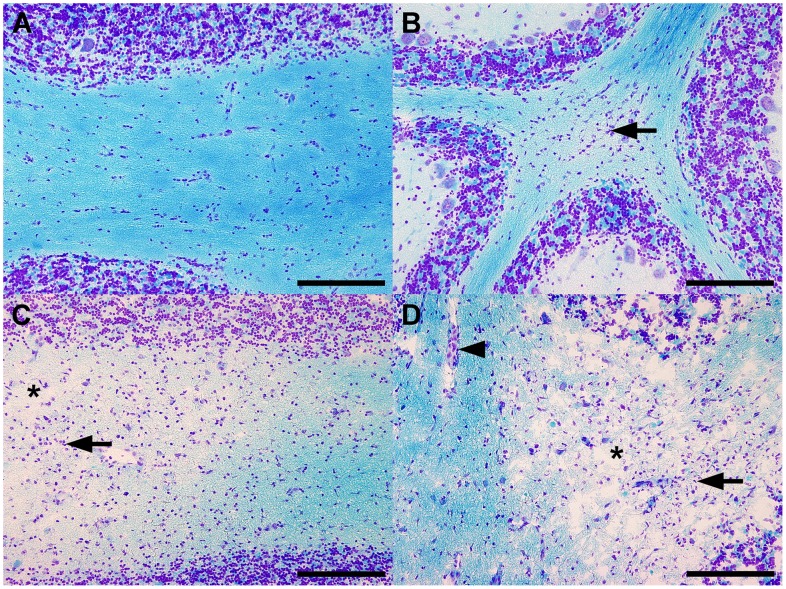
Pathohistological changes characteristic for the different subtypes of CDV leukoencephalitis. (**A**) The cerebella of the non-infected control dogs (group 1) displayed no histological alterations. (**B**) The cerebella of dogs affected by acute CDV leukoencephalitis (group 2) showed focal astro- and microgliosis (arrow) and occasionally few vacuolated myelin sheaths. (**C**) The cerebella of dogs affected by subacute CDV leukoencephalitis with demyelination but without inflammation (group 3) exhibited focally demyelinated white matter (asterix) combined with astro- and microgliosis (arrow). (**D**) The cerebella of dogs affected by chronic CDV leukoencephalitis with demyelination and with inflammation (group 4) displayed focally demyelinated white matter (asterix), combined with astro- and microgliosis (arrow) as well as perivascular inflammatory infiltrates (arrowhead). Luxol fast blue-cresyl violet. Scale bars = 200 µm.

Dogs were categorized into four groups based on the results of CDV nucleoprotein immunohistochemistry and histological changes as observed in following sections taken from the frozen tissue specimens used for RNA extraction. Accordingly, none of the control dogs exhibited CDV nucleoprotein immunoreactivity, whereas focal to multifocal CDV nucleoprotein-positive lesions were detectable in the white matter of the cerebellum and brain stem of all CDV-infected dogs (data not shown). The control dogs revealed no Luxol fast blue-negative white matter and no gliosis and/or inflammatory infiltrates within the cerebellum and brain stem (Figure 1A). The focal to multifocal lesions in the dogs with acute CDV leukoencephalitis were characterized by slightly increased numbers of astrocytes and microglia (Figure 1B). Additionally, the lesions in the dogs with subacute CDV leukoencephalitis exhibited loss of Luxol fast blue-positive white matter, indicating demyelination (Figure 1C). The lesions in the dogs with chronic CDV leukoencephalitis displayed loss of Luxol fast blue-positive white matter and perivascular, lymphohistioplasmacytic infiltrates (Figure 1D).

### Top-down Analysis of Major transcriptional changes in CDV Leukoencephalitis

**Figure 2:**
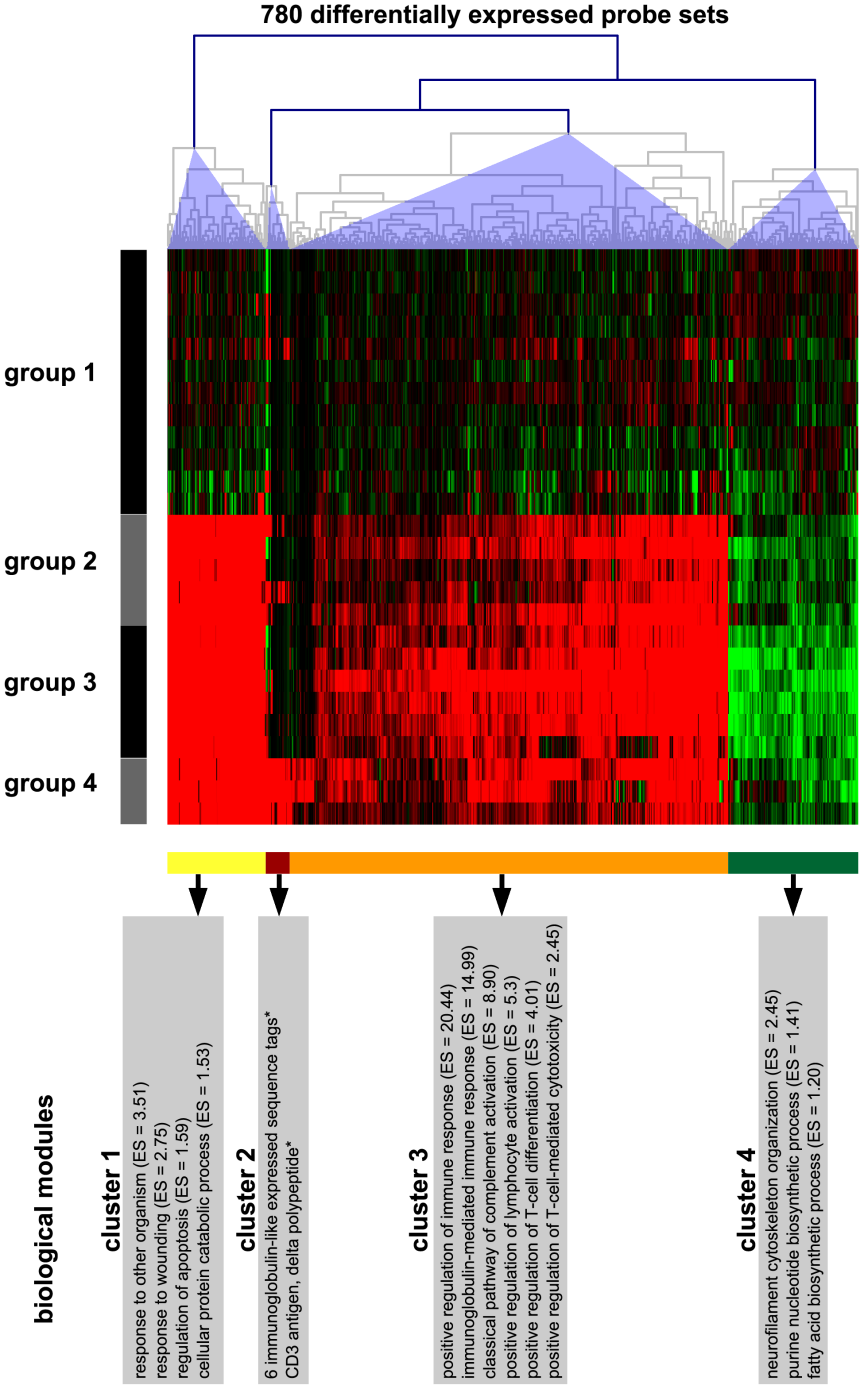
Heatmap displaying the expression profile of the 780 DEPs. Hierarchical cluster analysis of the 780 DEPs was performed employing Euclidean distance and complete linkage to reveal similar expression patterns. Each column represents one of the 780 DEPs and each row one of the 26 biological replicates (cerebellar specimens of individual dogs) sorted according to the histologically defined subtypes of CDV leukoencephalitis: group 1 = controls; group 2 = acute CDV leukoencephalitis; group 3 = subacute CDV leukoencephalitis; group 4 = chronic CDV leukoencephalitis. The heatmap displays the log_2_-transformed individual fold changes relative to the mean expression of the controls indicated by a color scale ranging from –2 (4-fold down-regulation) in green to 2 (4-fold up-regulation) in red. Accordingly, the DEPs are subdivided into four clusters with distinct expression profiles (yellow, brown, orange and green bars below the heatmap). Gene ontology analysis of the orthologous mouse genes followed by functional annotation clustering revealed distinct biological modules within the DEPs of cluster 1, 3, and 4. * = important associated genes and expressed sequence tags are reported for the DEPs of cluster 2 since automated gene ontology analysis revealed no significant results due to poor annotation quality. ES = enrichment score.

This study was performed in order to gain an assumption-free overview of the major transcriptional changes in CDV leukoencephalitis. An initial principal components analysis of the data sets revealed clearly separated clusters for controls and CDV-infected dogs, whereas the histologically distinct subgroups of CDV leukoencephalitis displayed some overlap, suggesting that CDV-infection is the dominant factor influencing the variance (Figure S1). One-factorial, multigroup analysis of differential expression employing LIMMA revealed 1374 probe sets with q≤0.05. Sequential filtering employing *post-hoc* pairwise tests with q≤0.05 and a fold change filter of ≥2.0 or ≤−2.0 revealed a total of 780 probe sets corresponding to 442 genes considered as differentially expressed in at least one of the six *post-hoc* comparisons (for a list of all 780 differentially expressed probe sets [DEPs], see Table S1). Hierarchical clustering of these DEPs revealed four clusters with a distinct expression profile (Figure 2). The first cluster included 111 DEPs (57 genes; Figure 2, cluster 1, yellow bar) and displayed a high up-regulation in all CDV-infected dogs. The second cluster consisted of 27 DEPs (16 genes; Figure 2, cluster 2, brown bar) and showed a high up-regulation in chronic CDV leukoencephalitis only. The third cluster included 495 DEPs (301 genes; Figure 2, cluster 3, orange bar) and displayed a moderate up-regulation in all CDV-infected dogs. Lastly, the fourth cluster comprised 147 DEPs (82 genes; Figure 2, cluster 4, green bar) and exhibited a moderate down-regulation in all CDV-infected dogs. Only few and less informative significantly enriched gene ontology terms were retrieved for the two clusters of probe sets highly and moderately up-regulated in all CDV-infected dogs employing canine probe set IDs as input in the gene ontology analysis (data not shown). In contrast, our alternative approach of using the orthologous mouse gene symbols revealed many significantly enriched gene ontology terms for the three clusters of probe sets highly and moderately up-regulated as well as moderately down-regulated in all CDV-infected dogs. Functional annotation clustering was performed to summarize these gene ontology terms into biological modules (Figure 2). The cluster of probe sets selectively and highly up-regulated in chronic CDV leukoencephalitis exhibited no significantly enriched gene ontology terms in the automated analysis due to insufficient annotation quality. Manual inspection revealed that the majority of the probe sets within this cluster (21 out of 27) were related to multiple *immunoglobulin-like expressed sequence tags*. Furthermore this cluster contained the *delta polypeptide of the cluster of differentiation 3* (*CD3) antigen (CD3D)*.

**Figure 3:**
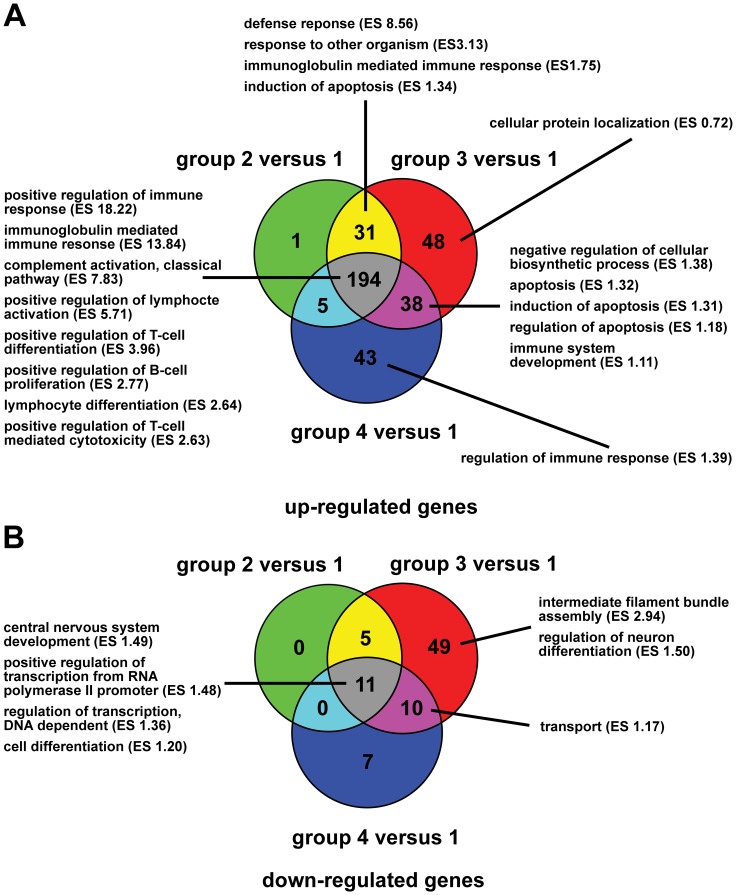
Venn diagrams comparing the DEGs within the *a priori* defined subtypes of CDV leukoencephalitis. The three subtypes of CDV leukoencephalitis (group 2–4) are compared for shared and unique DEGs as well as associated biological modules as detected by gene ontology analysis and functional annotation clustering of the orthologous mouse genes. (**A**) The Venn diagram displays the numbers and intersections of the up-regulated DEGs in the three subtypes of CDV leukoencephalitis (group 2–4) as compared to controls (group 1), respectively. (**B**) The Venn diagram displays the numbers and intersections of the down-regulated DEGs in the three subtypes of CDV leukoencephalitis (group 2–4) as compared to controls (group 1), respectively. ES = enrichment score; group 1 = controls; group 2 = acute CDV leukoencephalitis; group 3 = subacute CDV leukoencephalitis; group 4 = chronic CDV leukoencephalitis.

The results of all six pairwise *post-hoc* comparisons are summarized in Table 2. Accordingly, large numbers of differentially expressed genes (DEGs) were detected in the three *post-hoc* tests comparing the three subtypes of CDV leukoencephalitis with the control dogs. In contrast, only low to moderate numbers of DEGs were detected between the three subtypes of CDV leukoencephalitis (for a list of all 442 DEGs, see Table S2). More than half of the DEGs in the *post-hoc* tests comparing the CDV-infected- with the control dogs were equally up-regulated in all three subgroups of CDV leukoencephalitis and were mostly associated with an activation of the adoptive humoral immune response, followed by the cell-mediated immune response (Figure 3A). In contrast, the largest subset of down-regulated DEGs was observed in subacute CDV leukoencephalitis as compared to controls. These down-regulated DEGs were functionally related to the gene ontology terms “intermediate filament bundle assembly” and “regulation of neuron differentiation” (Figure 3B).

**Table 2:** Transcriptional differences between controls (group 1) and subtypes of CDV leukoencephalitis (group 2–4).

Pairwise*post-hoc*tests†	DEPstotal	DEPsup/down		DEGs‡ up/down	Biological modules§
**Group 2** **versus 1**	423	up	398	231	positive regulation of immune response (ES 19.71);immunoglobulin mediated immune response (ES 16.82);complement activation, classical pathway (ES 9.88);positive regulation of lymphocyte activation (ES 5.04);positive regulation of T-cell differentiation (ES 3.83);positive regulation of T-cell mediated cytotoxicity (ES 2.65);positive regulation of B-cell proliferation (ES 2.51)
		down	25	16	regulation of apoptosis (ES 2.10); positive regulationof apoptosis (ES 1.82); central nervous systemdevelopment (ES 1.30); positive regulation of transcriptionfrom RNA polymerase II promoter (ES 1.23)
**Group 3** **versus 1**	688	up	549	311	positive regulation of immune response (ES 18.94);immunoglobulin mediated immune response (ES 14.98);complement activation, classical pathway (ES 8.63);positive regulation of lymphocyte activation (ES 5.21);positive regulation of T-cell differentiation (3.95);positive regulation of T-cell mediated cytotoxicity (ES 2.42)
		down	139	75	intermediate filament bundle assembly (ES 2.82);fatty acid biosynthetic process (ES 1.28)
**Group 4** **versus 1**	525	up	486	280	immunoglobulin mediated immune response (ES 12.21);complement activation, classical pathway (ES 7.03);positive regulation of lymphocyte activation (ES 5.58);positive regulation of T-cell differentiation (ES 4.42);positive thymic T-cell selection (ES 3.15);positive regulation of T-cell mediated cytotoxicity(ES 2.65); positive regulation of B-cell proliferation(ES 2.45); positive regulation of NF-kappaBtranscription factor activity (ES 2.25)
		down	39	28	central nervous system development (ES 1.81);positive regulation of transcription from RNApolymerase II promoter (ES 0.82)
**Group 3** **versus 2**	106	up	70	44	protein amino acid N-linked glycosylation (ES 1.81);negative regulation of apoptosis (ES 1.57);regulation of lymphocyte proliferation (ES 1.49)
		down	36	21	intermediate filament bundle assembly (ES 3.33);nervous system development (ES 1.83);regulation of neuron differentiation (ES 1.70)
**Group 4** **versus 2**	89	up	77	47	anatomical structure development (ES 0.47);response to biotic stimulus (ES 0.43); regulationof immune response (ES 0.43); protein metabolicprocess (ES 0.17)
		down	12	8	immune response (ES 1.31); regulation ofbiological process (ES 0.26); cellularmetabolic process (ES 0.04)
**Group 4** **versus 3**	229	up	100	54	intermediate filament bundle assembly (ES 2.94);regulation of neuron differentiation (ES 1.27)
		down	129	75	complement activation, classical pathway (ES 2.32);protein amino acid N-linked glycosylation (ES 1.51)

† Multigroup test followed by independent pairwise *post-hoc* comparisons employing LIMMA with a false discovery rate (Benjamini and Hochberg) filter of q≤0.05, and a fold change filter of ≥2.0 or ≤ −2.0.

‡ Gene as defined by the DAVID knowledgebase. Multiple probe sets may match to a single gene, and probe sets may be unassigned (expressed sequence tags).

§ Biological modules associated with the DEG as detected by the DAVID functional annotation clustering algorithm using orthologous mouse genes.

DEGs = differentially expressed genes; DEPs = differentially expressed probe sets; ES = enrichment score; group 1 = control dogs, group 2 = acute CDV leukoencephalitis, group 3 = subacute CDV leukoencephalitis, group 4 = chronic CDV leukoencephalitis.

### Bottom-up Analysis of Transcriptional changes of Glial Marker Genes

The results of an independent analysis of the microarray data of manually selected glial cell marker genes employing classical statistical methods are shown in Table 3. Accordingly, there was a significant down-regulation of *myelin basic protein (MBP)*, *myelin-associated oligodendrocyte basic protein (MOBP)* and *proteolipid protein 1 (PLP1)*, and a similar trend for *2′,3′-cyclic nucleotide 3′ phosphodiesterase (CNP)*, *myelin associated glycoprotein (MAG)* and *myelin oligodendrocyte glycoprotein (MOG)* in subacute CDV leukoencephalitis as compared to controls. Additionally, *MBP* and *MOBP* were down-regulated in subacute CDV leukoencephalitis as compared to acute CDV leukoencephalitis. Interestingly, *MBP, MOBP* and *PLP1* gene expression revealed a significant reversal to normal control levels in chronic CDV leukoencephalitis as compared to subacute CDV leukoencephalitis. The oligodendroglial progenitor cell (OPC) marker genes *chondroitin sulfate proteoglycan 4 (CSPG4*, synonym: *NG2*) and *platelet-derived growth factor receptor, alpha polypeptide (PDGFRA)*, as well as the Schwann cell marker genes *myelin protein zero (MPZ,* synonym: *P0*) and *nerve growth factor receptor* (*NGFR*, synonym: *p75*) displayed no differential expression. Since the expression values of *CSPG4, MPZ,* and *NGFR* were clearly lower than the mean expression of the smallest 1.5 pM spiked-in BioB hybridization control RNA and displayed a constant value in all animals, it is assumed that the expression of these genes was below the detection sensitivity of the assay. There was a mild but significant down-regulation of the Schwann cell myelin-specific *peripheral myelin protein 22 (PMP22)* in acute and subacute CDV leukoencephalitis versus controls. The astrocyte marker gene *glial fibrillary acidic protein (GFAP)* displayed an up-regulation in all CDV-infected- versus control dogs.

**Table 3:** Analysis of microarray data of manually selected glial cell marker genes.

Gene Symbol	Kruskal-Wallis-test†	Group 2 versus 1†		Group 3 versus 1†		Group 4 versus 1†		Group 3 versus 2†		Group 4 versus 2†		Group 4 versus 3†	
	p-value	Fold change	p-value	Fold change	p-value	Fold change	p-value	Fold change	p-value	Fold change	p-value	Fold change	p-value
***Oligodendrocyte***													
*CNP*	0.214	−1.15	0.574	−1.58	0.067	1.08	0.734	−1.38	0.329	1.24	0.786	1.71	0.095
*MAG*	0.069	−1.43	0.442	−2.95	0.018	−1.24	0.233	−2.07	0.247	1.15	0.786	2.38	0.048
*MBP*	0.006	−1.29	0.383	−4.34	0.000	−1.14	0.365	−3.37	0.017	1.13	1.000	3.80	0.024
*MOBP*	0.005	−1.47	0.506	−7.26	0.000	−1.65	0.295	−4.93	0.030	−1.12	0.571	4.39	0.024
*MOG*	0.859	1.14	0.879	−1.19	0.820	1.56	0.448	−1.35	0.931	1.37	0.393	1.86	0.905
*PLP1*	0.028	1.17	0.879	−3.61	0.005	−1.18	0.633	−4.23	0.052	−1.38	0.571	3.06	0.024
***Oligodendroglial precursor cell***													
*CSPG4*	0.489	−1.00	0.646	−1.00	0.616	−1.00	0.734	1.00	1.000	1.00	1.000	1.00	1.000
*PDGFRA*	0.412	1.09	0.442	−1.36	0.250	−1.13	0.945	−1.48	0.177	−1.23	0.393	1.20	0.548
***Schwann cell***													
*MPZ*	0.343	1.00	1.000	1.00	0.616	1.00	1.000	1.00	0.662	1.00	1.000	−1.00	0.714
*NGFR*	1.000	1.00	1.000	1.00	1.000	1.00	1.000	1.00	1.000	1.00	1.000	1.00	1.000
*PMP22*	0.003	−1.50	0.002	−1.40	0.007	−1.09	0.536	1.07	0.329	1.39	0.036	1.29	0.095
***Astrocyte***													
*GFAP*	0.006	2.07	0.037	1.89	0.003	2.32	0.004	−1.10	0.931	1.12	0.786	1.23	0.714

† Kruskal−Wallis-test and independent pairwise *post-hoc* Mann-Whitney U-tests; group 1 = control dogs, group 2 = acute CDV leukoencephalitis, group 3 = subacute CDV leukoencephalitis, group 4 = chronic CDV leukoencephalitis.

CNP = 2′,3′-cyclic nucleotide 3′ phosphodiesterase; CSPG4 = chondroitin sulfate proteoglycan 4; GFAP = glial fibrillary acidic protein; MAG = myelin associated glycoprotein; MBP = myelin basic protein; MOBP = myelin-associated oligodendrocyte basic protein; MOG = myelin oligodendrocyte glycoprotein; MPZ = myelin protein zero; NGFR = nerve growth factor receptor; PDGFRA = platelet-derived growth factor receptor, alpha polypeptide; PMP22 = peripheral myelin protein 22; PLP1 = proteolipid protein 1.

### Exploratory Analysis of Pathways Correlated to Demyelination

**Figure 4:**
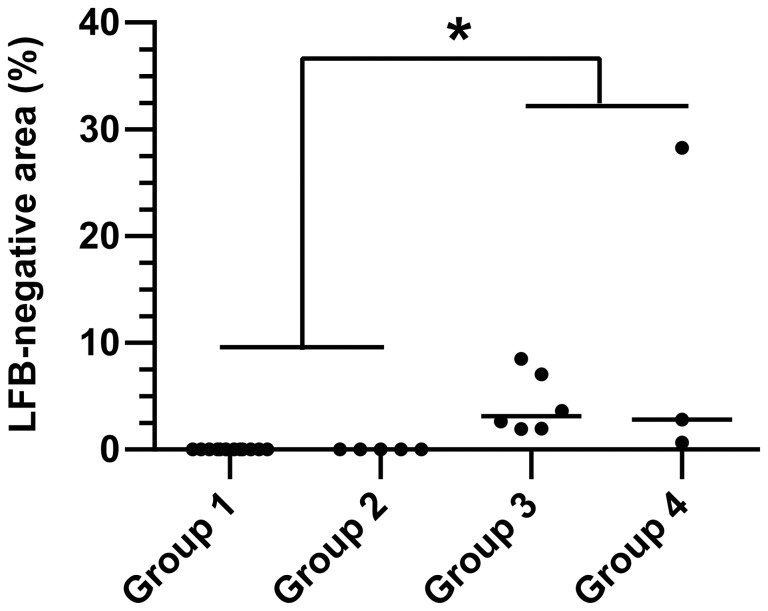
Multifocal demyelination is the hallmark of subacute and chronic CDV leukoencephalitis. The dot diagram shows an increased percentage of Luxol fast blue-negative white matter area (myelin loss) in subacute CDV leukoencephalitis (group 3) and chronic CDV leukoencephalitis (group 4) as compared to controls (group 1) and acute CDV leukoencephalitis (group 2). Each point represents the percentage of Luxol fast blue-negative area relative to total white matter area within the cerebellar specimen of each individual dog. The median of each group is represented by a horizontal line. Significant differences between the groups as revealed by the Kruskal-Wallis test with independent pairwise *post-hoc* Mann-Whitney U-tests are marked as follows: *p≤0.05.

In order to identify biological processes intimately related to demyelination, Gene Set Enrichment Analysis was performed employing Pearson’s correlation coefficient as metric to rank the genes according to their correlation to myelin-loss as observed in Luxol fast blue-cresyl violet stained slides. As already reported above, histology revealed a consistent multifocal loss of Luxol fast blue-positive white matter within subacute and chronic CDV leukoencephalitis. However, due to the variable proportion of the area affected by the multifocal demyelinated lesions as compared to the normal appearing white matter area present in each individual specimen, the percentage of Luxol fast blue-negative white matter area relative to the total white matter area present on sections directly following the tissue used for RNA extraction was calculated as input for Gene Set Enrichment Analysis. Accordingly, subacute and chronic CDV leukoencephalitis exhibited a significantly increased percentage of demyelinated white matter area as compared to controls and acute CDV leukoencephalitis (Figure 4). Gene Set Enrichment Analysis revealed six gene ontology terms positively correlated to demyelination and one gene ontology term negatively correlated to demyelination (Table 4). A Leading Edge Analysis revealed that the three gene ontology terms “viral reproduction”, “viral infectious cycle”, and “viral reproductive process”, which were all positively correlated with demyelination, can be summarized into a cluster based on the shared genes *chemokine (C-C motif) ligand 2 (CCL2), CD81 molecule (CD81)*, and *topoisomerase (DNA) II alpha 170kDa (TOP2A)* (Figure S2). The gene ontology term “generation of precursor metabolites and energy”, which was negatively correlated to demyelination included the down-regulated genes *D-aspartate oxidase (DDO), protoporphyrinogen oxidase (PPOX), glycogen synthase kinase 3 beta (GSK3B), aldehyde dehydrogenase 5 family, member A1 (ALDH5A1),* and *cytochrome c oxidase copper chaperone (COX17)*.

**Table 4:** Gene ontology terms correlated to demyelination as revealed by Gene Set Enrichment Analysis.

Gene ontology terms(biological process)	Size(N)	Gene symbols	Normalizedenrichmentscore	p-value
**Gene ontology ** ***terms positively correlated with*** ** Luxol fast blue** ***-negative white matter area (myelin-loss)***				
Viral reproduction	5	*CALCOCO2, CD81, TOP2A, CCL2, HBXIP*	1.71	0.0099
Viral infectious cycle	4	*CD81, TOP2A, CCL2, HBXIP*	1.65	0.0224
Viral reproductive process	4	*CD81, TOP2A, CCL2, HBXIP*	1.65	0.0224
Mitotic cell cycle	9	*TPX2, KIF23, CDKN1A, BTG3, SMC4, NEK6, CDKN2C, AFAP1L2, CDK2AP1*	1.62	0.0358
Humoral immune response	4	*BLNK, PSMB10, CCL2, LY86*	1.58	0.0106
Regulation of protein metabolic process	7	*TIMP1, IRAK3, CD81, TLR3, DNAJC1, CCND2, TLR1*	1.43	0.0395
**Gene ontology ** ***terms negatively correlated with*** ** Luxol fast blue** ***-negative white matter area (myelin-loss)***				
Generation of precursor metabolites and energy	5	*DDO, PPOX, GSK3B, ALDH5A1, COX17*	−1.30	0.0396

CDKN2C = cyclin-dependent kinase inhibitor 2C; AFAP1L2 = actin filament associated protein 1-like 2; ALDH5A1 = aldehyde dehydrogenase 5 family, member A1; BLNK = B-cell linker; BTG3 = BTG family, member 3; CALCOCO2 = calcium binding and coiled-coil domain 2; CCL2 = chemokine (C-C motif) ligand 2; CCND2 = cyclin D2; CD81 = CD81 molecule; CDK2AP1 = CDK2-associated protein 1; CDKN1A = cyclin-dependent kinase inhibitor 1A; COX17 = cytochrome c oxidase, subunit XVII assembly protein homolog; DDO = D-aspartate oxidase; DNAJC1 = DnaJ (Hsp40) homolog, subfamily C, member 1; GSK3B = glycogen synthase kinase 3 beta; HBXIP = hepatitis B virus x interacting protein; IRAK3 = interleukin-1 receptor-associated kinase 3; KIF23 = kinesin family member 23; LY86 = lymphocyte antigen 86; NEK6 = NIMA (never in mitosis gene a)-related kinase 6; PPOX = protoporphyrinogen oxidase; PSMB10 = proteasome (prosome, macropain) subunit, beta type, 10; SMC4 = structural maintenance of chromosomes 4; TIMP1 = tissue inhibitor of metalloproteinase 1; TLR1 = toll-like receptor 1; TLR3 = toll-like receptor 3; TOP2A = topoisomerase (DNA) II alpha 170 kDa; TPX2 = TPX2, microtubule-associated, homolog.

### Detection of CDV Nucleoprotein Employing Microarray Analysis, RT-qPCR and Immunohistochemistry

**Figure 5:**
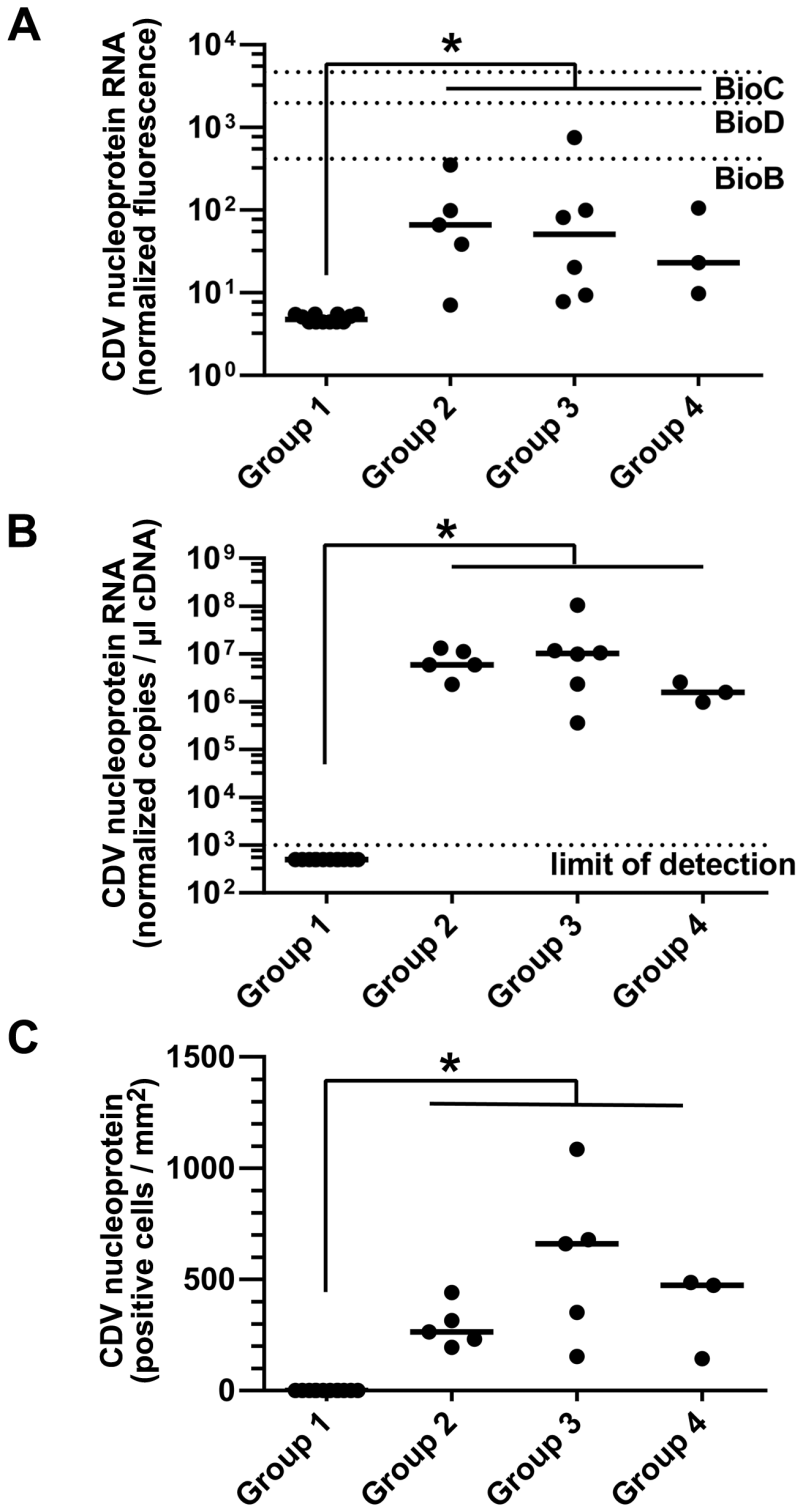
Detection of CDV nucleoprotein by microarray analysis, RT-qPCR and immunohistology. (**A**) Microarray analysis showed a low to moderate normalized CDV *nucleoprotein* RNA fluorescence signal in all CDV-infected dogs (group 2–4), which significantly exceeded the fluorescence signal measured in control dogs (group 1). BioB = mean expression of 1.5 pM *Escherichia coli biotin synthase (bioB)* spiked-in hybridization control RNA; BioC = mean expression of 5 pM *Escherichia coli biotin biosynthesis protein (bioC)* spiked-in hybridization control RNA; BioD = mean expression of 25 pM *Escherichia coli dethiobiotin synthetase (bioD)* spiked-in hybridization control RNA. (**B**) RT-qPCR demonstrated robust normalized CDV *nucleoprotein* RNA copy numbers in the cerebella of all CDV-infected dogs (group 2–4), whereas the control dogs (group 1) were negative. (**C**) Immunohistology revealed a significantly increased density of CDV nucleoprotein antigen-positive cells within the lesions of all CDV-infected dogs (group 2–4), whereas the cerebella of the control dogs (group 1) lacked a CDV-specific signal. (**A–C**) Each point represents the cerebellar specimen of an individual dog. The median of each group is represented by a horizontal line. Significant differences between the groups as revealed by the Kruskal-Wallis test with independent pairwise *post-hoc* Mann-Whitney U-tests are marked as follows: *p≤0.05.

The canine genome 2.0 array contains seven probe sets targeting different RNAs of the CDV (Table S1). In order to verify the microarray results, the data obtained by the probe set targeting the *CDV nucleoprotein* were compared to findings obtained by RT-qPCR and immunohistology. Accordingly, the probe set targeting the *CDV nucleoprotein* RNA revealed low to moderate normalized fluorescence values in all CDV-infected dogs, which were significantly exceeding those of the controls (Figure 5A). RT-qPCR revealed high normalized *CDV nucleoprotein* RNA copy numbers in all CDV-infected dogs as compared to no expression in controls (Figure 5B). In contrast, expression of the house-keeping gene *GAPDH* was detected in all dogs and confirmed successful RNA isolation and cDNA synthesis (data not shown). As already mentioned above, the quantitative analysis of the density of intralesional CDV nucleoprotein-immunoreactive cells displayed increased numbers of CDV nucleoprotein-positive cells within the lesions of all CDV-infected dogs, whereas the controls were consistently negative (Figure 5C). Correlation analysis revealed a significant, high, positive correlation of the log_10_-transformed *CDV nucleoprotein* RNA expression as measured by microarray analysis and RT-qPCR (Pearson’s correlation coefficient, r = 0.772, p<0.001). The CDV nucleoprotein antigen expression as measured by immunohistochemistry revealed a significant, moderate to high, positive correlation to *CDV nucleoprotein* RNA expression as determined by microarray analysis (Pearson’s correlation coefficient, r = 0.510, p<0.009) and RT-qPCR (Pearson’s correlation coefficient, r = 0.698, p<0.001), respectively.

## Discussion

### Top-down Analysis of Major Transcriptional changes in CDV Leukoencephalitis

The transcriptional changes within all CDV leukoencephalitis subtypes were dominated by up-regulated genes related to the gene ontology terms “immunoglobulin-mediated immune response” and “complement activation, classical pathway”. Furthermore, hierarchical cluster analysis revealed a cluster of DEPs highly up-regulated in chronic CDV leukoencephalitis only. The majority of these DEPs were specific for multiple *immunoglobulin-like expressed sequence tags*. These findings support previous descriptions of an intrathecal immunoglobulin synthesis and deposition in the inflammatory phase of CDV leukoencephalitis [14], [29]. The demonstration of significant amounts of anti-myelin autoantibodies by Vandevelde et al. (1986) substantiates our hypothesis that locally produced autoantibodies and complement may be involved in the pathogenesis of demyelination in the chronic inflammatory subtype of CDV leukoencephalitis [29], similar to Theiler’s murine encephalomyelitis [47].

Another remarkable group of genes up-regulated in all CDV leukoencephalitis subtypes was associated with the gene ontology term “positive regulation of T-cell-mediated cytotoxicity”. This finding is in agreement with the diffuse infiltration of the CNS by CD8-positive lymphocytes as observed by immunohistochemistry in all subtypes of CDV leukoencephalitis [48], [49]. Whether the cytotoxic T-cell reaction in CDV leukoencephalitis is solely directed against CDV antigens or also spreads to autoantigens in later phases is currently unknown.

The down-regulation of genes related to the gene ontology terms “intermediate filament bundle assembly” and “regulation of neuron differentiation” in subacute CDV leukoencephalitis points towards a possible involvement of neurons and/or axons in the pathogenesis of CDV leukoencephalitis. Notably, infection of neurons is thought to be a transient feature of early CDV leukoencephalitis [6], [18]. Furthermore, the observed down-regulation of neurofilaments is in agreement with previous studies showing progressive axonal pathology and loss beginning in the acute to subacute subtype of CDV leukoencephalitis [32]. Whether, these changes are a feature of primary axonal degeneration (inside-out model) or a response to primary demyelination leading to secondary axonal degeneration (outside-in model), needs to be elucidated in further studies [32], [50].

### Bottom-up Analysis of Transcriptional changes of Glial Cell Marker Genes

The observed significant down-regulation of myelin proteins like *MBP, MOBP* and *PLP1*, and a trend towards lower expression of *CNP*, *MAG*, and *MOG* in subacute CDV leukoencephalitis only is substantiated by immunohistological studies describing a loss of MBP and MAG in experimentally-induced CDV leukoencephalitis starting 16–21 days post infection and prior to the onset of perivascular inflammation [51]. Comparably, loss of MBP immunoreactivity is described in subacute and chronic lesions but not acute plaques in naturally-occuring CDV leukoencephalitis [52]. *In vitro* studies using CDV-infected mixed brain cell cultures indicate that demyelination is either directly mediated by a restricted infection of oligodendrocytes [23], or indirectly triggered by CDV-induced changes in other glial cells [53]. No matter whether the initiating event is directly or indirectly CDV-induced, *in vivo* as well as *in vitro* studies suggest that a metabolic impairment of oligodendrocytes leading to down-regulation of myelin gene synthesis is the main mechanism of demyelination in the initial phase of CDV leukoencephalitis, preceding oligodendrocyte loss by a slow and non-apoptotic “dying back”-mechanism [54], [55]. The apparent discrepancy between a rebound of myelin gene expression to control levels in chronic CDV leukoencephalitis despite ongoing demyelination suggests that myelin-loss is mediated by other mechanisms in the inflammatory phase of the disease, supporting the idea of a biphasic mode of demyelination in CDV leukoencephalitis [56].

### Exploratory Analysis of Pathways Correlated to Demyelination

Gene Set Enrichment Analysis was applied to identify pathways intimately associated with demyelination. Accordingly, the three gene ontology terms with the highest positive correlation to demyelination were related to gene expression changes known to be induced in the host organism by virus replication. This finding is supported by the present and previously performed spatiotemporal studies of CDV RNA and protein expression in CDV leukoencephalitis, showing that the presence of CDV is a prerequisite for the sequential development of demyelination [16]. The up-regulated genes shared by all three gene ontology terms were *CCL2, CD81*, and *TOP2A*, and represent highly interesting candidate genes concerning the initial steps of CDV-mediated demyelination. CCL2 (synonym: MCP-1) is a pro-inflammatory chemokine known to be produced by macrophages, T-cells, fibroblasts, keratinocytes, endothelial cells and astrocytes that attracts and activates monocytes [57], [58]. A toll-like receptor 3 (TLR3)-dependent up-regulation of CCL2 has been demonstrated for Theiler’s murine encephalomyelitis virus-infected astrocytes *in vitro* [58]. Furthermore, the observation of increased disease severity in Theiler’s murine encephalomyelitis virus-infected transgenic mice overexpressing CCL2 under the control of a GFAP promoter highlights a possible role of virus-induced, astrocyte-derived CCL2 in the pathogenesis of demyelination [59]. Notably, TLR3, known to recognize dsRNA associated with viral infections [57], was also one of the up-regulated genes in the present study and is included in the gene ontology term “regulation of protein metabolic process”.

The gene ontology term “generation of precursor metabolites and energy”, which was the only gene set negatively correlated to demyelination is defined in the gene ontology database to include “chemical reactions and pathways resulting in the formation of precursor metabolites, substances from which energy is derived, and any process involved in the liberation of energy from these substances” [41]. In detail, it comprised the genes *D-aspartate oxidase (DDO)*, a peroxisomal flavoprotein that catalyzes the oxidative deamination of D-aspartate and N-methyl D-aspartate, *protoporphyrinogen oxidase (PPOX)* involved in heme biosynthesis, the serine-threonine kinase *glycogen synthase kinase 3 beta (GSK3B)* participating in energy metabolism and neuronal cell development, the mitochondrial succinic semialdehyde dehydrogenase *aldehyde dehydrogenase 5 family, member A1 (ALDH5A1)* that plays a role in GABA-metabolism, and *cytochrome c oxidase copper chaperone (COX17)*, a non-structural subunit of the mitochondrial respiratory chain cytochrome c oxidase [60]. Whether some or all of these genes which were collectively down-regulated in CDV leukoencephalitis are involved in the suggested initial metabolic dysfunction of oligodendrocytes needs to be addressed in future studies.

In summary, the transcriptional changes in CDV leukoencephalitis support the concept of a biphasic mode of demyelination with a presumably initial non-apoptotic oligodendrocyte dystrophy, followed by a second wave of an intrathecally synthesized immunoglobulin- and complement-mediated autoimmunity.

## Supporting Information

Figure S1 **Principal components analysis of all data sets.** The clearly separated clusters of control- and CDV-infected dogs suggest a robust and systematic difference in their cerebellar transcriptomes.
(TIF)

Figure S2 **Leading Edge Analysis of the gene ontology terms significantly correlated to demyelination.** The Leading Edge Analysis revealed that multiple gene ontology terms involved in the host’s response to viral replication can be summarized into one cluster based on shared genes.
(TIF)

Table S1 **780 probe sets differentially expressed in one or more of the pairwise** ***post-hoc*** **tests.** All 43035 probe sets of the GeneChip canine genome 2.0 array (Affymetrix) were analyzed for differential expression between controls (group 1) and the three subtypes of CDV leukoencephalitis (group 2–4) employing a multigroup LIMMA test followed by six pairwise *post hoc* tests with q≤0.05 and a fold change filter of ≥2.0 or ≤−2.0. The table shows the probe set IDs, gene symbols, gene titles according to the Affymetrix annotation file (release 33; 29. October 2012), fold-changes, p-values, q-values, and an assignment to the corresponding cluster shown in Figure. 2.
(XLSX)

Table S2 **442 genes differentially expressed in one or more of the pairwise** ***post-hoc*** **tests.** The list of 780 DEPs was consolidated to a list of 442 DEGs. Genes were defined according to the integrative DAVID knowledgebase (DAVID ID) [38]. This table displays the DAVID IDs, gene titles according to the DAVID knowledgebase, the fold-changes, p-values and q-values of the multigroup LIMMA test and six pairwise *post hoc* tests, and an assignment to the corresponding Venn diagram subset shown in Figure 3.
(XLSX)
