# Supplementary figures and images for: Transcriptional Changes in Canine Distemper Virus-Induced Demyelinating Leukoencephalitis Favor a Biphasic Mode of Demyelination
Source: PLoS One. 2014 Apr 22;9(4):e95917. doi: 10.1371/journal.pone.0095917 (PMC3995819; doi:10.1371/journal.pone.0095917)

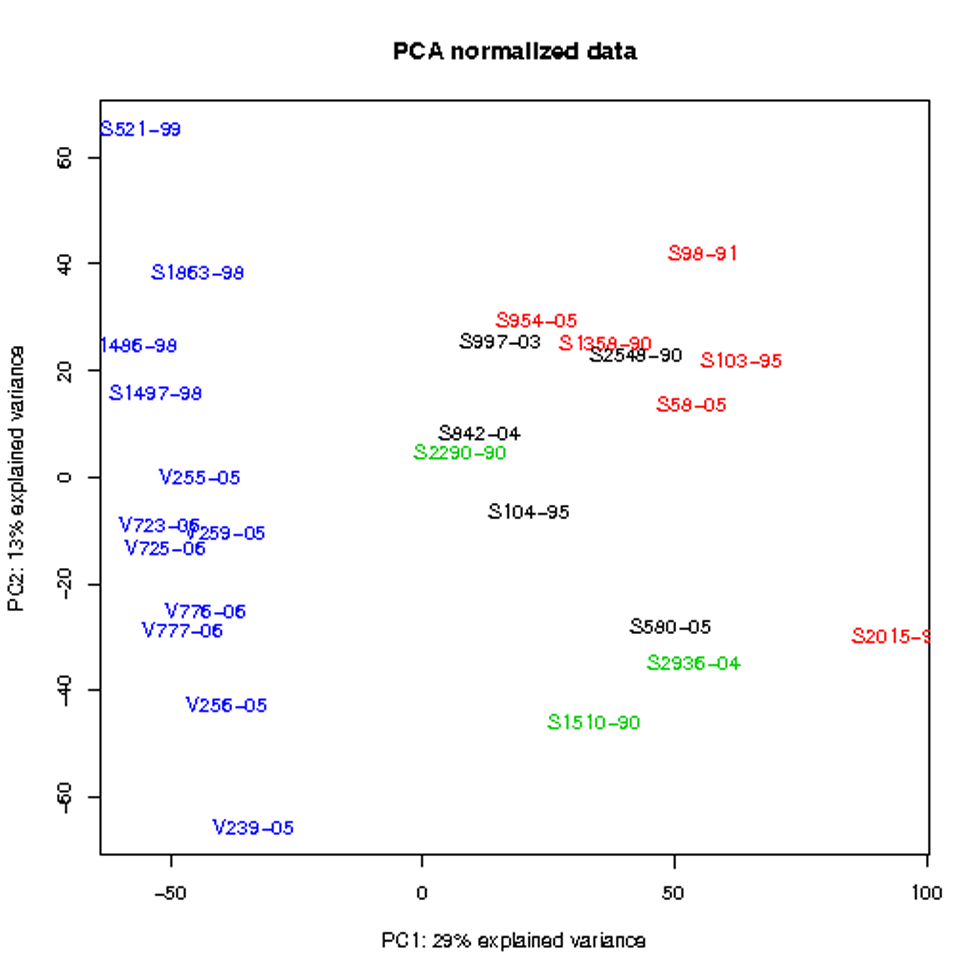

Supplement: Figure S1 — Principal components analysis of all data sets. The clearly separated clusters of control- and CDV-infected dogs suggest a robust and systematic difference in their cerebellar transcriptomes. (TIF) [file pone.0095917.s001.tif]

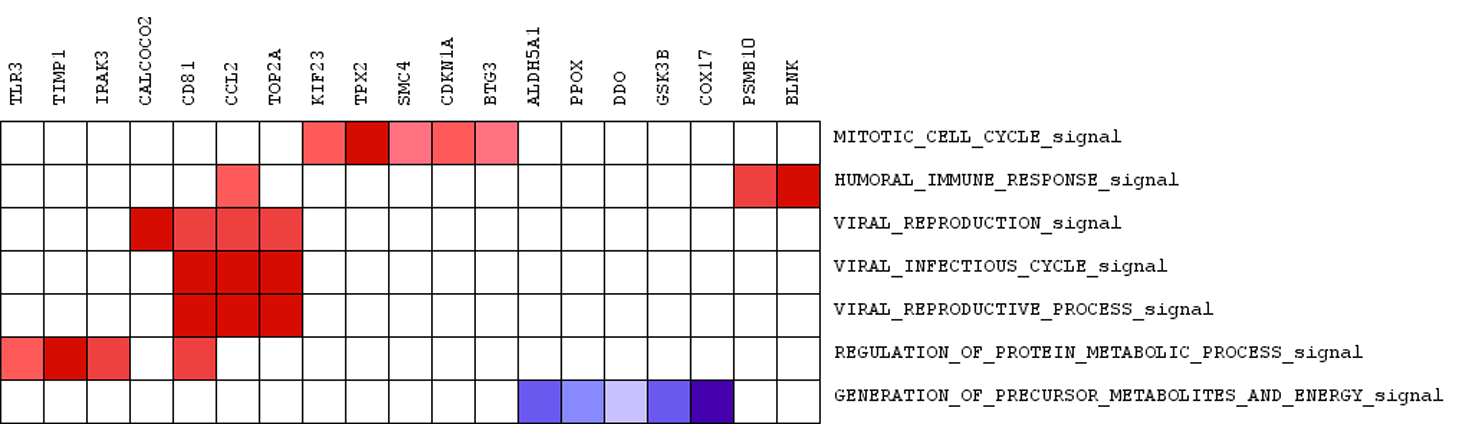

Supplement: Figure S2 — Leading Edge Analysis of the gene ontology terms significantly correlated to demyelination. The Leading Edge Analysis revealed that multiple gene ontology terms involved in the host’s response to viral replication can be summarized into one cluster based on shared genes. (TIF) [file pone.0095917.s002.tif]
